# Indications for and Risks Associated With Implant Removal After Pediatric Trauma

**DOI:** 10.5435/JAAOSGlobal-D-22-00050

**Published:** 2022-04-15

**Authors:** Blake K. Montgomery, James G. Gamble, Stephanie T. Kha, Garin G. Hecht, John S. Vorhies, Justin F. Lucas

**Affiliations:** From the Department of Orthopaedic Surgery, Santa Clara Valley Medical Center, San Jose, CA (Dr. Lucas and Dr. Hecht); Stanford Orthopaedics, San Jose, CA (Dr. Lucas and Dr. Hecht); and the Department of Orthopaedic Surgery, Stanford University, Redwood City, CA (Dr. Montgomery, Dr. Gamble, Dr. Kha, and Dr. Vorhies).

## Abstract

A wide range of implants are used in the treatment of pediatric fractures, including wires, plates, screws, flexible rods, rigid rods, and external fixation devices. Pediatric bones differ from adult bones both mechanically and biologically, including the potential for remodeling. Implants used in pediatric trauma patients present a unique set of circumstances regarding indications, risks, timing of implant removal, weight-bearing restrictions, and long-term sequelae. Indications for implant removal include wire/pin fixation, when substantial growth remains, and infection. When considering implant removal, the risks and benefits must be assessed. The primary risk of implant removal is refracture. The timing of implant removal varies widely from several weeks to a year or more with the option of retention depending on the fracture, type of implant, and skeletal maturity of the patient.

Pediatric fractures are one of the most common injuries in childhood. Although many pediatric fractures can be managed nonsurgically, certain fracture characteristics make surgical management with internal fixation preferable. Many implants are routinely used in the surgical treatment of these injuries, including wires, screws, plates, and flexible and rigid rods. Although there is widespread agreement regarding application of most implants, evidence-based guidelines to determine when and whether the implants should be removed do not currently exist.^1-3^ Independent factors including practice philosophy, patient age and compliance, cost factors, and availability of surgical resources has made determining whether and when pediatric implants should be removed a topic of great interest and debate in the orthopaedic community.

One of the main reasons implants in pediatric patients have received special attention is because of the growth potential of pediatric patients. Unlike in adult patients, surgeons have to consider how the implant will affect the growing bone and how the growing bone might affect the implant.

Allergies, metal corrosion, carcinogenesis, and interference with future arthroplasty are common areas of interest with retained implants. Approximately 17% of women and 3% of men are allergic to nickel and 1% to 3% are allergic to chromium.^4^ Stainless steel contains nickel and chromium. Despite the relatively common nature of metal allergies, allergic reactions leading to adverse effects to orthopaedic implants are uncommon.^5^ Most of the allergy research in orthopaedic surgery has focused on arthroplasty. Allergic reactions to metal occur primarily through T-cell delayed-type hypersensitivity (type IV). Cutaneous reaction to metal does not seem to correlate with adverse effects of implantation. One prospective study determined that a small number of patients had a cutaneous metal allergy, but no deep allergic symptoms or adverse effects after implantation with a 2-year follow-up.^6^

Because metal implants electrochemically interact with aqueous human body solution, the metal may undergo slow corrosion. Corrosion may lead to increased concentration of trace metal within body tissues.^7^ Abnormal levels of metal ions in patients with spinal implants have been reported; however, there were not any accompanying adverse clinical effects.^8^ Corrosion from implants in fracture care is less common in comparison with that in arthroplasty. This may be due to the differing functions of fracture implants and less motion at fracture sites in comparison with a large amount of motion within joints after arthroplasty.

Only a handful of case reports have reported malignant tumors occurring in the region of orthopaedic implants.^1,9^ These reports are very rare, and most are after arthroplasty. One case series reported 12 cases of sarcoma associated with multiple different orthopaedic implants.^9^ Three of the patients were younger than 20 years at the time of implantation. Two of those patients had femoral staples, and one had a femoral nail for fracture. Tumor occurrence near implants used in fracture care is extremely rare.

As patients age, inevitably, some patients will develop arthritis of the hip or knee. Patients with retained hip implants undergoing implant removal at the time of total hip arthroplasty are at greater risk of requiring a revision femoral stem, intraoperative fracture, and needing bone graft (Figure 1).^10^ Overall, when an implant is present, subsequent arthroplasty surgery is considered much more complex than standard total hip arthroplasty. Total knee arthroplasty in the setting of retained implant is understudied. One retrospective study found that patients who had previous implants at the time of total knee arthroplasty were more likely to have mechanical complications after total knee arthroplasty.^11^

**Figure 1:**
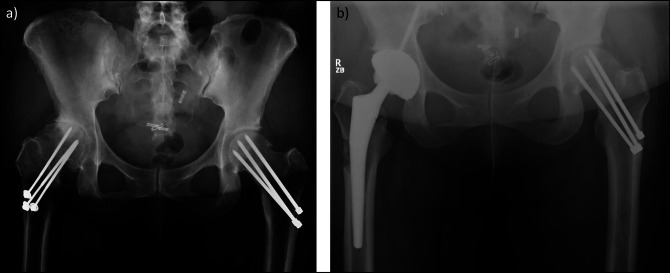
**A**, Adult AP pelvis radiograph showing retained Knowles pins in bilateral hips for the treatment of slipped capital femoral epiphysis 40 years earlier. **B**, AP pelvis radiograph after total hip arthroplasty requiring a significant amount of bone to be removed from the lateral cortex of the femur to allow for the removal of the pins.

## Common Indications for Implant Removal

The indications for implant removal after pediatric trauma can be relative indications or absolute indications. Each clinical scenario is unique, and implant removal must be assessed in the context of growth remaining, fracture healing, and the risks of implant removal. Typical indications that may warrant removal include migration of the implant, infection, exposed implants, symptomatic implants, when notable growth remains, concern for future complications, or electively at the patient's requests.

## Current American Academy of Orthopaedic Surgeons Clinical Practice Guidelines and Appropriate Use Criteria

The American Academy of Orthopaedic Surgeons provides a synthesis of recommendations based on reviews of high-quality evidence and expert consensus on many subjects throughout orthopaedic surgery. Both supracondylar humerus fractures and diaphyseal femur fractures have been thoroughly reviewed with evidence-based recommendations provided.^2,3^ No current recommendation for the timing of pin removal could be made for supracondylar humerus fractures. Similarly, for pediatric femur fractures, no current recommendation exists for or against implant removal because of the lack of evidence.

## Pins/Wires

Pins and wires are some of the most common implants used for pediatric fracture fixation. They can be smooth or threaded and are typically made of stainless steel. The malleable nature of smaller wires allows them to be easily bent and/or cut to the appropriate length. Some of the most used sizes are 1.6 mm (0.062 inches) and 2.0 mm (5/64 inches). By convention, diameters below 2 mm are called Kirschner wires while diameters of 2 mm and above are Steinmann pins, although in practice, the terms pin and wire are often used interchangeably. They are used in many anatomic locations and often inserted in a percutaneous fashion. In areas where it is necessary to cross an open physis with internal fixation, the smooth nature of wires is thought to be less likely to cause a physeal arrest affecting future growth.^12^ Common areas for fixation with pins or wires include supracondylar humerus fractures, distal radius fractures, and displaced Salter-Harris type 1 distal femur physeal fractures. Wires can be buried deep into the skin or can be left exposed. They typically serve as temporary fixation through the initial course of fracture healing and are subsequently removed once inherent bony stability has been achieved.

Absolute indications for pin or wire removal are infection and healed fractures with exposed wires, while relative indications for pin or wire removal include wire migration, symptomatic implant such as soft-tissue irritation, implant breakage, and patient or family preference.

Although wires maintain fracture fixation well, several adverse events can occur. Exposed wires can lead to a pin site infection or superficial infection.^13^ Rarely, these wires can lead to osteomyelitis or septic arthritis.^14^ One retrospective study evaluated pin site infections in patients with supracondylar humerus fractures.^14^ They reported a 3.1% superficial infection rate and 1.2% deep infection rate (osteomyelitis or septic arthritis). In both distal femur and distal humerus fractures, percutaneously placed pins are often intra-articular. Prolonged retention of intra-articular pins may theoretically increase the risk of a small pin-site infection leading to septic arthritis. One recent study of percutaneous pin fixation of the distal femur found that the overall incidence of pin tract infection was 6.7%, and retention of pins for more than 4 weeks was associated with an increased risk of pin tract infections. No cases of septic arthritis were reported in this cohort of 163 patients.^15^ In the setting of a superficial pin site infection, the pins are usually removed and the patient can be started on a course of oral antibiotics at the discretion of the surgeon.

In addition to infection, other adverse events such as wire migration can be an indication for removal depending on where the wire has migrated and which structures are at risk.^13^ Migration can refer to movement or loosening of the wire at the fracture site; however, remote migration of wires into locations such as the thoracic cavity has been reported as well.^16^ Wire migration can injure the surrounding soft tissues (nerve, blood vessels, muscles, and tendons) and can penetrate the skin. If there is little concern for soft-tissue injury or if migration is minimal and the fracture is still being adequately held, the wires are often retained and monitored closely. Wires are often removed early if there is concern for soft-tissue injury or if a buried wire has penetrated the skin.

Wire site irritation can also be problematic and can be an indication for removal. This can occur with either exposed or buried wires. In certain anatomic regions, irritation can be localized to a specific tendon or nerve, such as the ulnar nerve in the case of a medial pin in the distal humerus or the extensor pollicis longus tendon in the case of the distal radius. Tendon irritation can often be managed with close monitoring. Nerve irritation can be an indication for early pin removal.

Pin failure or breakage in situ is not frequently reported. If this occurs, the broken pin is commonly left in situ if it cannot be easily retrieved, is not crossing the physis, is not intra-articular, and is not at risk for causing soft-tissue injury.

The timing of wire removal usually occurs around 3 to 4 weeks, with a few exceptions.^17^ There is not enough high-quality research to make strong evidence-based recommendations determining the best time of pin or wire removal. Our clinical consensus is to remove pins and wires in approximately 3 to 4 weeks for distal humerus fractures. In general, extra-articular fractures treated with closed reduction are appropriate for pin removal at 3 weeks, whereas intra-articular fractures, fractures treated in older patients, and those treated with open reduction may warrant retention of wires for 4 weeks or longer in select cases. Pediatric patients with femur fractures treated with wire and pin fixation typically undergo pin removal in 4 to 6 weeks depending on the age of the patient and open versus closed reduction. We advise close monitoring, particularly in the setting of intra-articular pins retained for longer than 3 to 4 weeks.

Exposed pins can often be removed in the outpatient clinic setting and is well tolerated in children.^17^ One study assessed pain levels in pediatric patients undergoing pin removal in the clinic after fixation of supracondylar humerus fractures.^17^ They assessed 47 patients and found that the children experienced little or no pain during this in-office procedure. Buried pins are traditionally removed in the operating room.

## Plates/Screws

There are many different types of screws and plates used in pediatric fractures. Screws are often made of stainless steel but can be made of titanium as well. Depending on the location and fracture morphology, screws can be cortical or cancellous, fully or partially threaded, and locking or nonlocking. Screws can be applied independent of a plate or within a plate. Washers can be used in certain pediatric fractures as well, for example, in medial epicondyle fractures or physeal fractures with a large Thurston-Holland fragment. Plates are typically made of stainless steel or titanium. The plate type can vary but can include tubular plates, dynamic compression plates, and locking plates.

An absolute indication for plate or screw removal is infection, whereas relative indications for plate or screw removal include healed fractures, malunion or nonunion, screw migration, symptomatic implant including soft-tissue irritation, implant breakage, and patient or family preference.

**Figure 2:**
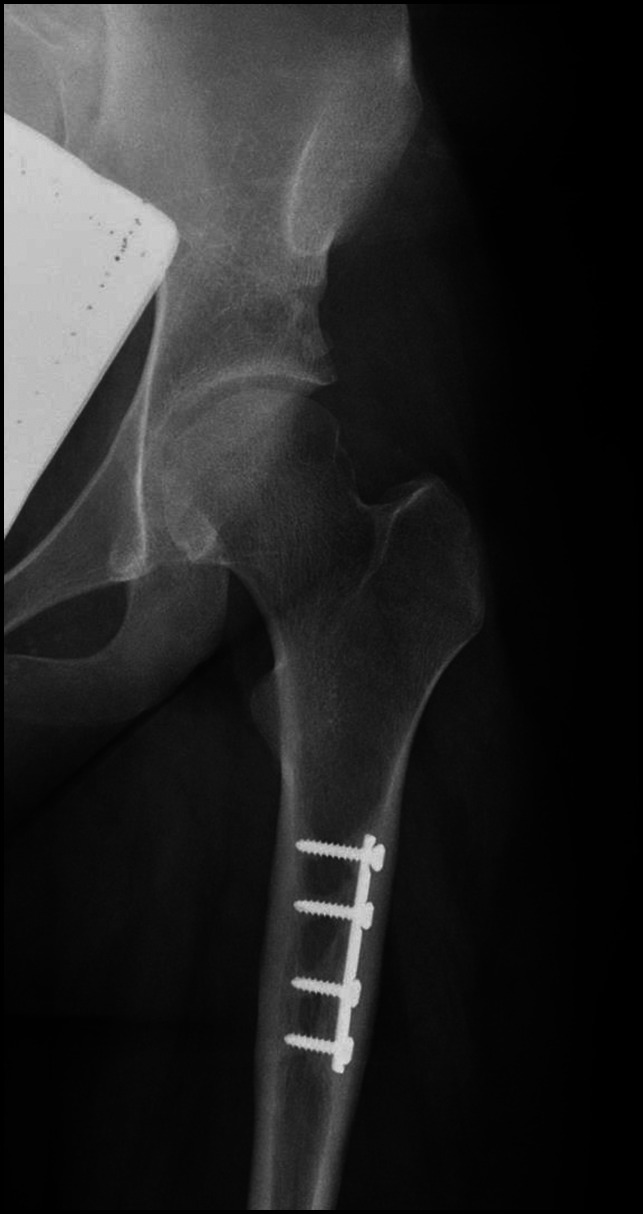
AP radiograph of the left hip of a 19-year-old female patient showing bone overgrowth of a retained plate.

Risk is associated with both screw and plate removal as well as for retention of these implants. First, regarding retention, the surgeon and the family have to consider the possible long-term adverse effects. The longer an implant is in place, the more likely it becomes that the bone can grow over the plate and screws, making these implants more difficult to remove later on (Figure 2).^18,19^ One retrospective study of implant removal after submuscular plating examined 22 patients and determined that bone overgrowth was associated with increased difficulty of plate removal.^19^

Retained plates can also create a stress shielding effect on the underlying bone leading to osteopenia. A retrospective case series assessed complications due to plate retention after submuscular plating in pediatric femur fractures.^18^ They found that three patients had stress shielding at the edge of the plate and two of the three patients even underwent a CT scan for additional assessment of the stress shielding.

**Figure 3:**
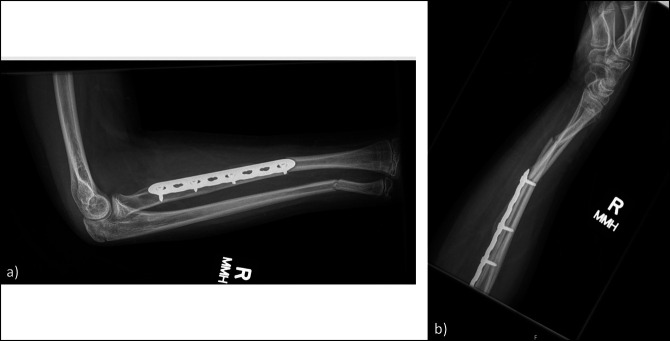
**A**, AP and (**B**) lateral radiograph of the right forearm of a 14-year-old boy demonstrating periprosthetic both-bone forearm fracture adjacent to the retained plate on the radius. The plate was placed 1 year earlier.

Fractures can occur adjacent to the retained implant (Figure 3).^20-22^ In one study of 83 patients younger than 13 years who underwent plate fixation for a forearm fracture and had implants retained for a minimum of 2 years, the authors found six implant-related refractures.^22^ One of the fractures occurred within 1 year of original fixation, while the other five associated fractures occurred between 19 and 34 months after fixation. Removing the implant, however, may also predispose a patient to an increased risk of refracture. In a study of 82 children (younger than 13 years) with 112 plates removed from the forearm, the authors found 11 refractures after the removal of the plate within 1 year of implantation.^23^ Controversy remains in the literature on whether there is a notable difference in refracture risk between patients with retained implants and patients with removed implants.

If notable growth remains, plates may lead to angular deformity or growth arrest. Angular deformity has been well described in the femur, even when fixation does not cross the physis.^18,24^ One retrospective case series assessed complications after lateral plate fixation of pediatric femur fractures.^24^ There were 85 patients who underwent plate fixation. Two patients developed distal femoral valgus deformity and subsequently underwent corrective osteotomy and implant removal. Not surprisingly, screws that cross a growing physis have high potential to cause growth disturbance. This is particularly relevant in the proximal femur where threaded implants for fixation of femoral neck fractures are placed across the proximal femoral physis.^25^

**Figure 4:**
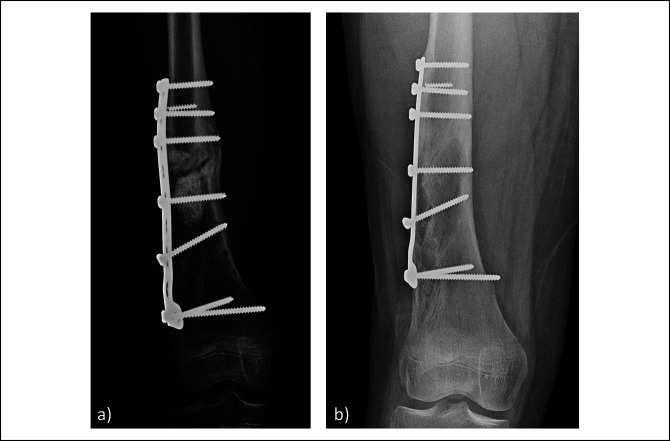
**A**, AP radiograph of the right distal femur of a 12-year-old boy demonstrating revision surgical fixation of a pathologic fracture. **B**, AP radiograph of the right distal femur 2 years later showing the cutback phenomenon of the most distal screw. Significant growth of the distal femur has occurred compared with the previous radiograph. The most distal screw that was previously in the metaphysis is now metadiaphyseal and more prominent.

Retained implants can also become symptomatic and may become infected, which may be indications for removal. Screws that were once in the bone may protrude into soft tissues causing local irritation. This phenomenon has been specifically documented in the growing child when screws are placed across the metaphysis. As the bone grows, the screw tips are often exposed through cutback because of the funnelization process when the growing metaphysis becomes the diaphysis (Figure 4).^26^ Implants can also serve as a nidus for late deep infection.^27^ Although the list of possible long-term effects is plentiful, the overall rate of any adverse event occurring with retained implants is actually quite low.^21^

Removing screws and plates is not a benign procedure and carries risks that should be discussed preoperatively with the patient and family. During removal, there is a risk of damaging neurovascular structures. Screws can strip or break, and some aspect of the implant may be left behind. Larger or even new incisions may be required to remove the screw or plate.^19^ This is especially true if there is bone overgrowth of the implant. In addition, the procedure to remove the implant can be as long as or longer than the index surgery.^1^ The surgery itself can lead to infection.^21,28^ The removal of the implant can also make patients susceptible to refracture due to the aforementioned phenomenon of stress shielding.^28^ One systematic review of 871 patients from 10 case series found a 10% complication rate associated with implant removal, and complications reported included fracture, infection, hematoma, wound dehiscence, and failure to remove the entire implant.^1^

The risks of removing implants can also be specific to the anatomic location, particularly in the hip. Implant removal around the hip can lead to osteonecrosis of the femoral head in patients who may have avoided this complication if the implant was retained.^29^ One retrospective study of 371 pediatric patients with proximal femur fractures who underwent implant removal found that three patients developed osteonecrosis after implant removal.^29^ This study delineated other risks associated with implant removal—including osteonecrosis and refracture, delayed wound healing, and nerve damage—and found a total complication rate of 14% in their study population.^29^

Furthermore, the hospitalization costs associated with the removal of implants in the pediatric population are not insignificant, with one study calculating implant-removal procedures over the course of 1 year accounting for a total of $131.3 million in-hospital charges nationally.^27^ This database study investigated 1,141 pediatric patients undergoing removal of implants in 2012 and found that admissions related to implant removal accounted for a mean length of stay of 2.9 days in the hospital, with average hospital charges amounting to $36,349 per admission ($21,040 when excluding infection-related removal).^27^ Providers should be aware of the direct costs associated with implant removal and the indirect costs, such as time away from school for the patient or time away from work for the caregiver, when considering implant removal procedures.

Symptomatic implants can also be a common, although relative, indication for removal.^20^ However, symptoms such as pain related to the implant can vary based on the anatomic region, plate size, or implant prominence. Palpable, subcutaneous plates on the ulna or the medial distal tibia may be easier to implicate as a source of discomfort than a proximal femoral plate for example. Nevertheless, improvement in pain symptoms after implant removal cannot be guaranteed. One study assessed pain scores and health-related quality of life in 11 pediatric patients who underwent routine implant removal.^30^ Seven patients had pain before removal of implants, and 43% still reported pain after removal, although most patients had normal health-related quality of life before and after removal.

Late infection can develop and can become an indication for implant removal and antibiotic treatment.^29^ Broken implants are a relative indication for removal, especially in the setting of malunion/nonunion or risk of migration. Occasionally, patients want implants removed for personal or cultural reasons. Screws and plates should be removed in asymptomatic patients if they request removal and understand the risks.

The appropriate time for screw and plate removal is not well established. The literature reports removal any time from 8 to 14 months; however, some authors electively remove implants up to 3 years from index surgery.^20,28,31^ When the decision is made to remove the implant instead of retaining it, our clinical consensus is to remove it at 12 months.

## Flexible Rods

Flexible intramedullary rods offer effective fracture fixation in many different types of long bone fractures. These implants are made of either titanium or stainless steel. Some rods include a locking option. Some surgeons use end caps on the proud portion of the rod while others do not. Flexible rods are often buried beneath the skin, but some surgeons leave the ends exposed when used in the forearm.^32^

An absolute indication for flexible rod removal is infection, whereas relative indications for flexible rod removal include healed fracture, nonunion or malunion, rod migration, symptomatic implant such as soft-tissue irritation, implant breakage, and patient or family preference.

Reports of retaining and removing flexible rods are available in the literature. The risk profile is different depending on which route is chosen. If implants are retained, then bone overgrowth can occur, which can make the rods much more difficult to remove later on.^33^ One study assessed 163 patients who underwent flexible rod removal. They found that one rod was unobtainable because of intramedullary migration and bone overgrowth at the insertion site.^33^ Patients can also have persistent discomfort in the region of the flexible rod.^34^ One study reported that a quarter of patients with retained flexible rods underwent an additional procedure because of persistent discomfort in the area.^34^ Retained implants may also be associated with infection.^33^ Retention of the flexible rods does not seem to contribute to leg length discrepancy or growth deformity.^35^

Elective removal of flexible rods is more commonly reported in the literature compared with retention.^36^ Multiple adverse events can occur when these implants are removed. During the removal operation, implants can break or can be challenging to retrieve. An infection can occur related to the removal surgery.^33^ In addition, refracture can occur through the unprotected bone. One retrospective study of 203 pediatric patients who underwent intramedullary nailing for forearm fracture reported that five patients had refracture after the implant was removed.^37^ Contracture of the adjacent joint has also been reported.^33^

**Figure 5:**
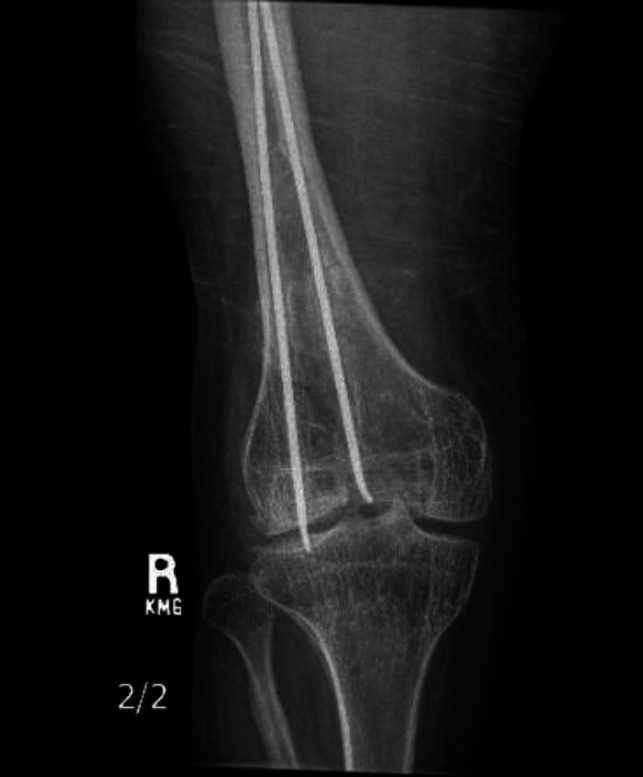
AP radiograph showing the right distal femur of a 30-year-old male patient with retained flexible nails that have migrated into the knee.

Multiple clinical signs and symptoms can be an indication for flexible rod removal. Migration of flexible rods can occur and can damage the surrounding skin and soft-tissue structures (Figure 5).^32^ These implants have a proud end that extends beyond the cortex and can cause soft-tissue irritation, which can be quite bothersome to patients.^38^ Implants associated with infection should be removed and antibiotics initiated.^33^ Implants can break, which warrants at the very least closer monitoring and possible removal, especially in the setting of malunion or nonunion.

The ideal time of flexible rod removal is currently not evidence-based. Our opinion is that flexible rods can be safely removed after fracture union, which may occur at different rates for children of different ages. Exposed rods in the forearm are generally removed sooner than the buried ones, similar to percutaneous pins, 1 to 2 months versus 3 to 6 months, respectively.^32^ Many surgeons remove flexible rods in the femur anywhere from 6 to 12 months after insertion if the fracture has healed as expected.^33,36,38^ Our clinical consensus is to remove buried rods in the forearm or humerus 3 to 12 months postoperatively and rods in the femur 6 to 18 months postoperatively.

## Rigid Nails

Rigid nails have a unique role in pediatric fracture surgery. They can be used to treat diaphyseal femur fractures, typically in patients approaching adolescence. Nails are typically antegrade, trochanteric entry, reamed, and statically locked. Lateral entry nails are preferred over piriformis entry nails because of decreased association of developing osteonecrosis.^39^ Rigid nails are not used in tibia fractures with widely open proximal physes, given the theoretical risk of the late development of recurvatum, although this is likely not an issue in older adolescents.^40^ These implants are made of stainless steel or titanium.

An absolute indication for rigid rod removal is infection, whereas relative indications for rigid rod removal include rod migration, symptomatic implant such as soft-tissue irritation, implant breakage, and patient or family preference.

The risks of retention of rigid implants are similar to implants previously mentioned. Overgrowth can occur, making these implants more challenging to remove. Implants can irritate the soft-tissue as the child grows and the bone remodels.^29^ There is also a risk that retained implants could serve as a nidus for infection.^29^ Asymmetric coxa valga has been reported in association with trochanteric entry rigid nails, related to premature arrest of the greater trochanteric apophysis or potentially stimulation of the proximal femoral physis.^41^ It is unclear that implant removal is able to mitigate this risk.

There is inherent risk-associated removal of rigid nails. A buried implant may be challenging to remove, leading to increased blood loss, increased size of surgical incision, and longer surgical times. The implant may break, and part of the implant may not be retrievable. In the postoperative period, an infection may arise or refracture may occur.

Many clinical situations may be an indication for rigid rod removal. Pain or irritation in the area is a common relative indication for removal, although pain improvement cannot be guaranteed. Irritation from rigid nails typically occurs around the interlocking screws or potentially at the proximal tip of the nail if it is proud beyond the greater trochanter. A deep infection should prompt removal and initiation of antibiotics.^41^ Broken implants should warrant assessment for the cause of the break and determine whether the implant poses any new soft-tissue risk. In the fracture setting, broken implants may also suggest nonunion; thus, an appropriate workup to determine the cause of the broken implants is essential.

We currently do not have high-quality studies to make an evidence-based decision regarding whether implants should be removed and, if so, the best time for elective implant removal. Reports in the literature suggest that elective implant removal is safe around 1 year after implantation.^42^ Because of the rarity of reported complications from retained rigid femoral nails, an alternative strategy is to consider only partial implant removal. Proud distal interlocking screws can become symptomatic because of cutback related to growth or the initial position. In cases where interlocking screws are placed in the proximal nail, the distal interlocking screws alone can be removed.^41^ Our clinical census is that if there is an indication for implant removal, it is best offered any time after fracture union but before substantial overgrowth occurs (6 months to 2 years).

## Summary

In summary, there are many indications for implant removal in pediatric patients. When retention is an option, the decision to retain or remove the implant is often a shared decision between the patient and the surgeon. If patients request elective implant removal, the risks of removal must be discussed and understood. In addition, the cost of elective implant removal is not always covered by insurance, and the family should have an understanding of the cost before the surgery.
